# Risk Factors, Management, and Follow-Up of Obstetric Anal Sphincter Injuries: An Audit From a UK District General Hospital

**DOI:** 10.7759/cureus.92426

**Published:** 2025-09-16

**Authors:** Anushae A Akhtar

**Affiliations:** 1 Internal Medicine, University Hospital Coventry and Warwickshire, Coventry, GBR

**Keywords:** anal sphincter injury, episiotomy, maternal morbidity, perineal trauma, postpartum complications, preventative measures, quality improvement (qi), rcog guidelines

## Abstract

Background: Obstetric anal sphincter injuries (OASIs) are a major cause of maternal morbidity. This audit evaluated risk factors, intrapartum management, postoperative care, and follow-up of OASI cases at a UK district general hospital, benchmarking practices against national guidance.

Methodology: Retrospective audit of women who sustained OASI between January 1 and December 31, 2023. Data were extracted from electronic and paper records. Outcomes were assessed against Royal College of Obstetricians and Gynecologists (RCOG) Green-top Guideline No. 29 standards. The prevalence of OASI among total and vaginal deliveries in 2023 was reported.

Results: Twenty-eight women sustained OASI; 23 (82%) were primiparous, and 10 (36%) had an instrumental delivery. Episiotomy was performed in 14 (50%). Perineal support was recorded in 19 (68%). Postoperative care aligned well with standards: antibiotics in 26 cases (93%), physiotherapy referral in 27 (96%), and laxatives in 22 (79%). Gynecology follow-up referral was documented in 21 (75%) cases. OASI prevalence in 2023 was 13.3% of all births and 22.8% of vaginal births at our unit.

Conclusions: Primiparity and instrumental delivery were common risk factors. Immediate postoperative care met most standards, but documentation and follow-up referrals were inconsistent. A structured OASI proforma and standardized referral pathway are recommended to improve continuity of care. Recognition of high-risk women and consistent use of preventive strategies, together with structured counselling for future deliveries, may further improve outcomes.

## Introduction

Obstetric anal sphincter injuries (OASIs) encompass third- and fourth-degree perineal tears and contribute substantially to short- and long-term morbidity. They are classified as Grade 3a (≤50% of external anal sphincter (EAS) thickness torn), Grade 3b (>50% EAS), Grade 3c (EAS and internal anal sphincter (IAS)), and Grade 4 (EAS, IAS, and anorectal mucosa). Complications include anal incontinence, perineal pain, dyspareunia, and psychological distress, all of which can affect quality of life [1,2].

In the United Kingdom, OASI incidence is typically reported around 2%-3% of vaginal births and higher among primiparas [3]. Established risk factors include nulliparity, instrumental delivery, fetal macrosomia, malposition (e.g., occipitoposterior), prolonged second stage, and certain maternal characteristics [4,5]. National guidance from the Royal College of Obstetricians and Gynecologists (RCOG) Green-top Guideline No. 29 emphasizes preventive measures (such as mediolateral episiotomy when clinically indicated during instrumental birth and perineal protection), optimal repair, postoperative antibiotics and laxatives, physiotherapy, and structured follow-up [6]. Occult anal sphincter injuries have also been described [7], and training programs have reduced OASI incidence in some settings [8].

Objectives

Primary

The primary objective was to evaluate intrapartum management, postoperative care, and follow-up of OASI cases at George Eliot Hospital in 2023, comparing practices against RCOG standards [6].

Secondary

The secondary objective was to describe maternal, obstetric, and fetal risk factors for OASI in this cohort and to report unit-level OASI prevalence [3-5].

## Materials and methods

Design and setting

This was a retrospective case-note audit conducted at George Eliot Hospital, UK.

Population

All women with third- or fourth-degree perineal tears between 1 January and 31 December 2023. Exclusions: incomplete notes or unclassified perineal trauma.

Data sources and variables

Data were obtained from BadgerNet and delivery suite records. Variables collected included demographics (age, race/ethnicity, parity); obstetric factors (mode of delivery, episiotomy, including angle where available, perineal support technique and feasibility); fetal factors (birthweight, percentile, head position at crowning if recorded, shoulder dystocia, prolonged second stage); postoperative management (antibiotics, laxatives, and bowel movement before discharge); and follow-up referrals (physical therapy, gynecology/specialist clinic). Where available, OASI grade (3a/3b/3c/4) was also recorded

Denominator data

In 2023, there were 211 births at our unit, of which 123 were vaginal (including 21 assisted vaginal) and 67 were by cesarean section. The OASI prevalence was 13.3% of all births and 22.8% of vaginal births.

Standards

Postoperative antibiotics, laxatives, physical therapy referral, and specialist follow-up were benchmarked against RCOG Green-top Guideline No. 29 [6].

Analysis

Descriptive statistics (counts and percentages) were used. Results were presented overall and, where helpful, by parity subgroup. Missing data were reported as *Not documented (ND)*.

Governance

The audit was registered (ID: 1422/922). All records were anonymized. Ethical approval was not required for this service evaluation.

## Results

Demographics and risk factors

Twenty-eight women sustained OASI. Of these, 23 were primiparous (82%) and 5 were multiparous (18%). Ethnicity was recorded as follows: United Kingdom/White European, 17; Albania, Czech Republic, or Romania, 3; Indian, African-Indian, or Sri Lankan, 2; Bengali, 1; Indian or Sri Lankan, 1; Other Asian, 1; Pakistani, 1 (Table 1).

**Table 1:** Demographics and obstetric factors (n = 28). OA, occipito-anterior; OP, occipito-posterior; OT, occipito-transverse; GA, gestational age

Variable	*n* (%)
Primiparous	23 (82)
Multiparous	5 (18)
Ethnicity	
White European	17 (61)
Albanian/Czech/Romanian	3 (11)
Indian	2 (7)
Bengali	1 (4)
Sri Lankan	1 (4)
Other Asian	1 (4)
Pakistani	1 (4)
Birthweight >4 kg	3 (11)
>90th centile	4 (14)
Small for GA	2 (7)
Shoulder dystocia	1 (4)
Fetal head position	
OA	20 (71)
OP	5 (18)
OT	1 (4)
Compound presentation	1 (4)
Not documented	1 (4)
Prolonged second stage	2 (7)

Fetal characteristics

Birthweight >4 kg was observed in 3/28 (11%) cases; >90th centile in 4/28 (14%); small for gestational age in 2/28 (7%); and shoulder dystocia occurred in 1/28 (4%). Fetal head position at delivery was occipito-anterior (OA) in 20, occipito-posterior (OP) in 5, occipito-transverse (OT) in 1, compound in 1, and not documented in 1 case. Two women experienced a prolonged second stage of labor.

Multiparous subgroup

This subgroup (*n* = 7, 27%) included both spontaneous and instrumental births, some of which involved previous episiotomy or perineal trauma. Their occurrence highlights that OASI is not limited to primiparous women and underscores the importance of counselling in future pregnancies.

Labor and delivery

Spontaneous vaginal birth occurred in 18/28 (64%) cases, while 10/28 (36%) were instrumental (forceps 6, vacuum 4). Episiotomy was performed in 14/28 (50%) overall, and in all instrumental births according to local practice. Perineal support was documented in 19/28 (68%) cases; a *hands-off* approach due to positioning was noted in 4/28 (14%), and not documented in 5/28 (18%). OASI grade distribution was: 3a = 9, 3b = 11, 3c = 4, and 4th degree = 4 (Table 2).

**Table 2:** Intrapartum management, postoperative care, and follow-up (n = 28). ND, not documented

Variable	*n* (%)
Instrumental birth	10 (36)
Episiotomy performed	14 (50)
Perineal support documented	19 (68)
Perineal support not feasible (positioning)	4 (14)
Perineal support ND	5 (18)
Antibiotics prescribed	26 (93)
Laxatives prescribed	22 (79)
Laxatives ND	6 (21)
Bowel opening recorded before discharge	19 (68)
Physiotherapy referral	27 (96)
Gynecology/specialist follow-up referral	21 (75)

Postoperative care and follow-up

Antibiotics were administered in 26/28 cases (93%), and laxatives in 22/28 (79%), with ND in 6 cases. Bowel opening before discharge was achieved in 19/28 (68%). Physiotherapy referral was made in 27/28 cases (96%), and gynecology or specialist follow-up referral in 21/28 cases (75%) [6].

## Discussion

This audit shows patterns consistent with the wider literature, including an increased risk of OASI among primiparas and following instrumental birth, as reported in published studies [3-5]. We also observed variation in preventive measures, such as perineal protection, which was partly dependent on documentation and, in some cases, limited by maternal positioning that restricted hands-on support.

Why do these patterns occur? Primiparity is associated with less distensible perineal tissues and higher instrumental rates [3,5]. Instrumental birth increases perineal strain; mediolateral episiotomy, when clinically indicated, may reduce severe trauma, but correct angle and execution are critical, as shown in previous reports [4]. Observed ethnic differences may reflect a combination of anatomic, biomechanical, and sociocultural factors; however, small numbers and potential confounding limit inference in our cohort.

Shoulder dystocia and macrosomia

Both shoulder dystocia and macrosomia are biologically plausible contributors to OASI [5]. In this audit, macrosomia (>4 kg) and dystocia were infrequent (3 and 1 cases, respectively), limiting statistical interpretation; nonetheless, we explicitly report them and recommend consistent capture due to their clinical relevance.

OASI prevalence compared with national data

The OASI prevalence in our unit (13.3% of all births, 22.8% of vaginal births) is higher than national averages (2%-6%) [3]. This may reflect the small denominator in our audit year, single-center design, and thorough case ascertainment. While the rate highlights the importance of continued vigilance and local quality improvement, caution is needed when comparing directly with larger population-based datasets.

Prolonged second stage

Two women had a prolonged second stage, another recognized risk factor for OASI, reinforcing the importance of timely decision-making in managing labor progression, consistent with other findings [5].

Multiparous subgroup (27%)

Although smaller, this group underscores that OASI is not confined to primiparas. We now describe their delivery characteristics to avoid under-emphasis and inform counselling in subsequent pregnancies, also supported by prior evidence [5].

Follow-up and longer-term outcomes

Late complications such as anal incontinence, dyspareunia, persistent perineal pain, and psychological distress are clinically important [1,2]. These were not systematically captured in this audit. We propose a re-audit at three to six months incorporating a standard symptom questionnaire (e.g., validated anal incontinence scale), physiotherapy attendance, and specialist clinic outcomes to quantify medium-term morbidity.

Prevention and high-risk identification

Although preventive measures were not formally audited, evidence supports strategies such as perineal massage in late pregnancy, the use of warm compresses during the second stage, and the correct technique of mediolateral episiotomy in instrumental deliveries [8]. Manual perineal support was documented in only 19/28 women (68%), yet it should ideally be offered universally where feasible. Recognition of high-risk women, particularly primiparas, those with prolonged second stage, instrumental births, macrosomic infants, or certain ethnic backgrounds, enables targeted preventive care [3-5]. Counselling about recurrence risk and mode of delivery in subsequent pregnancies, as well as structured postnatal follow-up of both physical and mental well-being, are important quality improvement priorities [6].

Quality improvement implications

A structured OASI proforma (covering OASI grade, episiotomy details including angle, perineal protection method/feasibility, fetal head position, second-stage duration, repair details, and mandated referrals) should reduce ND fields and standardize care. Embedding automatic referral orders (physiotherapy and specialist clinic) and discharge advice (antibiotics, laxatives, bowel care) may further improve adherence [6].

Strengths and limitations

Strengths include complete case capture and benchmarking against national standards [6]. Limitations are the small sample, retrospective design, and incomplete documentation (e.g., episiotomy angle). Lack of systematic medium-term outcomes constrains conclusions about longer-term morbidity [1,2].

## Conclusions

Primiparity and instrumental birth were common in OASI cases at our unit. Postoperative care largely met RCOG standards, but documentation and referral consistency require improvement. Implementing a structured proforma and standardized follow-up pathway should enhance continuity and outcomes. Reporting unit-level prevalence alongside case-level findings will aid longitudinal surveillance and benchmarking. In addition to these measures, recognition of high-risk women and consistent use of preventive strategies, together with structured counselling for future deliveries, may further improve outcomes.
